# Crystal structure of *trans*-di­chlorido­(1,4,8,11-tetra­aza­cyclo­tetra­decane-κ^4^ *N*)chromium(III) bis­(form­amide-κ*O*)(1,4,8,11-tetra­aza­cyclo­tetra­decane-κ^4^ *N*)chromium(III) bis­[tetra­chlorido­zincate(II)]

**DOI:** 10.1107/S2056989020004910

**Published:** 2020-04-09

**Authors:** Dohyun Moon, Jong-Ha Choi

**Affiliations:** a Beamline Department, Pohang Accelerator Laboratory, POSTECH, Pohang 37673, Republic of Korea; b Department of Chemistry, Andong National University, Andong 36729, Republic of Korea

**Keywords:** crystal structure, cyclam, chloride, formamide, *trans*-isomer, chromium(III) complex, synchrotron radiation

## Abstract

The structure of the title compound, [CrCl_2_(C_10_H_24_N_4_)][Cr(HCONH_2_)_2_(C_10_H_24_N_4_)][ZnCl_4_]_2_ (C_10_H_24_N_4_ = 1,4,8,11-tetra­aza­cyclo­tetra­decane, cyclam; HCONH_2_ = formamide, fa), has been determined from synchrotron X-ray data. The asymmetric unit contains two independent halves of the [CrCl_2_(cyclam)]^+^ and [Cr(fa)(cyclam)]^3+^ cations, and one tetra­chlorido­zincate anion. In each complex cation, the Cr^III^ ion is coordinated by the four N atoms of the cyclam ligand in the equatorial plane and two Cl ligands or two O-bonded formamide mol­ecules in a *trans* axial arrangement, displaying a distorted octa­hedral geometry with crystallographic inversion symmetry. The Cr—N(cyclam) bond lengths are in the range 2.061 (2) to 2.074 (2) Å, while the Cr—Cl and Cr—O(fa) bond distances are 2.3194 (7) and 1.9953 (19) Å, respectively. The macrocyclic cyclam moieties adopt the centrosymmetric *trans*-III conformation with six- and five-membered chelate rings in chair and *gauche* conformations*.* The crystal structure is stabilized by inter­molecular hydrogen bonds involving the NH or CH groups of cyclam and the NH_2_ group of coordinated formamide as donors, and Cl atoms of the ZnCl_4_^2−^ anion as acceptors.

## Abstract 2

In the title compound, [CrCl_2_(C_10_H_24_N_4_)][Cr(HCONH_2_)_2_(C_10_H_24_N_4_)][ZnCl_4_]_2_, the two Cr^III^ ions each show a distorted octa­hedral coordination with four N atoms of cyclam in the equatorial plane and two Cl^−^ anions or O-bonded formamide groups in axial positions. The macrocyclic moieties adopt the most stable *trans*-III conformation. In the crystal, extensive N—H⋯Cl and C—H⋯Cl hydrogen bonds connect the [CrCl_2_(C_10_H_24_N_4_)]^+^ and [Cr(HCONH_2_)_2_(C_10_H_24_N_4_)]^3+^ cations and tetra­chlorido­zincate anions, forming a three-dimensional network.

## Chemical context   

The 14-membered cyclam (1,4,8,11-tetra­aza­cyclo­tetra­decane, C_10_H_24_N_4_) has a moderately flexible structure, and its metal complexes can form either *trans* or *cis*-[*ML*_2_(cyclam)]^n+^ (*L* = a monodentate ligand) geometric isomers (Poon & Pun, 1980 ▸). Furthermore, the *trans* isomer can adopt five conformers, *viz. trans*-I (+ + + +), *trans*-II (+ − + +), *trans*-III (+ − − +), *trans*-IV (+ + − −) and *trans*-V (+ − + −), which differ in the chirality of the *sec*-NH centres (Choi, 2009 ▸), and where the plus sign indicates the hydrogen atom of the NH group is above the plane of the macrocycle and the minus sign indicates that it is below. The *trans*-I, *trans*-II and *trans*-V conformations can also fold to form *cis*-I, *cis*-II and *cis*-V conformers, respectively (Subhan *et al.*, 2011 ▸). Recently, it has been shown that cyclam derivatives and their metal complexes exhibit stem-cell mobilization and anti-HIV activity (Ronconi & Sadler, 2007 ▸; De Clercq, 2010 ▸; Ross *et al.*, 2012 ▸). The conformation of the macrocycle and the orientations of the N—H bonds in the complex are very important factors for co-receptor recognition. Therefore, knowledge of the conformation and the crystal packing in transition-metal compounds containing cyclam has become important in the development of new highly effective anti-HIV drugs (De Clercq, 2010 ▸). In addition, the formamide group can be coordinated to a metal ion through the oxygen or nitro­gen atoms (Balahura & Jordan, 1970 ▸). It should be noted that the geometric assignment and determination of the coordination mode based on spectroscopic properties is not always conclusive. We describe here the synthesis and structural characterization of a new double complex, [CrCl_2_(cyclam)][Cr(fa-O)_2_(cyclam)][ZnCl_4_]_2_, (I), which was performed to elucidate and confirm its mol­ecular structure unambiguously.
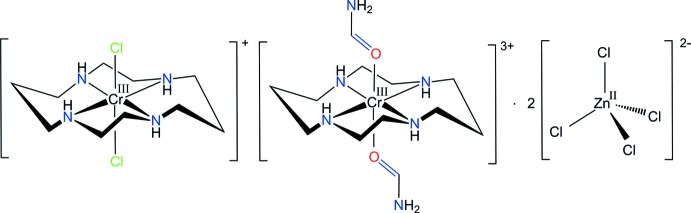


## Structural commentary   

**Figure 1:**
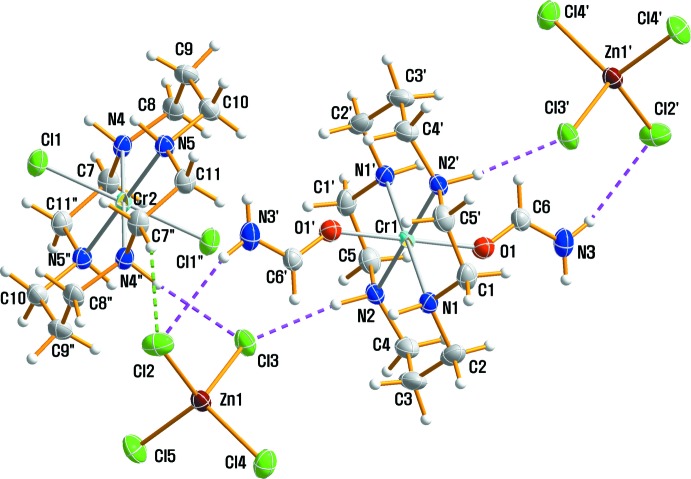
Mol­ecular structure of (I), drawn with displacement ellipsoids at the 50% probability level. The primed and double-primed atoms are related by symmetry operations (−*x* + 1, −*y* + 1, −*z* + 1) and (−*x* + 1, −*y* + 1, −*z*), respectively. Hydrogen bonds are shown as dashed lines.

Fig. 1 ▸ shows a displacement ellipsoid plot of (I) with the atom-numbering scheme. The crystallographic asymmetric unit of (I) is composed of two halves of independent [CrCl_2_(cyclam)]^+^ and [Cr(fa)(cyclam)]^3+^ cations and one tetra­chlorido­zincate anion. The two Cr atoms are located on crystallographic centers of symmetry, so these complex cations both have mol­ecular *C_i_* symmetry. Each cyclam moiety in the two Cr^III^ complex cations adopts the most stable *trans*-III conformation. The Cr^III^ ions are six-coordinated in a distorted octa­hedral geometry with the four N atoms of the macrocyclic ligand in equatorial positions and two Cl ligands or two O atoms of formamide mol­ecules in axial positions (Fig. 1 ▸). The Cr—N(cyclam) bond lengths are in the range 2.061 (2) to 2.074 (2) Å, in good agreement with those observed in *trans*-[Cr(ONO)_2_)(cyclam)]BF_4_ [2.064 (4)–2.073 (4) Å; De Leo *et al.*, 2000 ▸], *trans-*[Cr(NH_3_)_2_(cyclam)][ZnCl_4_]Cl·H_2_O [2.0501 (15)–2.0615 (15) Å; Moon & Choi, 2016*a* ▸], *trans-*[Cr(NCS)_2_(cyclam)]_2_[ZnCl_4_] [2.0614 (10)–2.0700 (10) Å; Moon *et al.*, 2015 ▸], *trans*-[Cr(NCS)_2_(cyclam)]ClO_4_ [2.046 (2)–2.060 (2) Å; Friesen *et al.*, 1997 ▸], *trans*-[Cr(nic-O)_2_(cyclam)]ClO_4_ [2.057 (4)–2.064 (4) Å; Choi, 2009 ▸], [Cr(ox)(cyclam)]ClO_4_ [2.062 (4)–2.085 (5) Å; Choi *et al.*, 2004*b* ▸], [Cr(acac)(cyclam)](ClO_4_)_2_·0.5H_2_O [2.065 (5)–2.089 (5) Å; Subhan *et al.*, 2011 ▸] and *cis*-[Cr(ONO)_2_(cyclam)]NO_2_ [2.0874 (16)–2.0916 (15) Å; Choi *et al.*, 2004*a* ▸]. However, the Cr—N bond lengths for the secondary amine of cyclam in the *trans* isomer are slightly shorter than those of the primary amine found in *trans*-[CrCl_2_(Me_2_tn)_2_]Cl [2.0861 (18)–2.1076 (18) Å; Choi *et al.*, 2007 ▸] and *trans*-[CrCl_2_(Me_2_tn)_2_]_2_ZnCl_4_ [2.0741 (19)–2.0981 (18) Å; Choi *et al.*, 2011 ▸]. The Cr—Cl and Cr–O (fa) bond lengths are 2.3194 (7) and 1.9953 (19) Å, respectively. The Cr—Cl distance is comparable to the values in *trans-*[CrCl_2_(cyclam)]Cl [2.3295 (6) Å; Solano-Peralta *et al.*, 2004 ▸], *trans-*[CrCl_2_(cyclam)]_2_[ZnCl_4_] [2.3472 (9) Å; Flores-Vélez *et al.*, 1991 ▸] and [CrCl_2_(cyclam)][Cr(ox)(cyclam)](ClO_4_)_2_ [2.3358 (14) Å; Moon & Choi, 2016*b* ▸]. As expected, the five-membered chelate rings adopt a *gauche* conformation, and the six-membered ring is in the chair conformation. The average bond angles of the five- and six-membered chelate rings around chromium(III) are 85.03 (9) and 94.97 (9)°, respectively. The uncoordinated ZnCl_4_^2−^ counter-anion remains outside the coordination sphere of the two Cr^III^ ions and has a distorted tetra­hedral geometry as a result of its involvement in hydrogen-bonding inter­actions. It exhibits Zn—Cl bond distances in the range 2.2555 (8) to 2.3035 (8) Å and Cl—Zn—Cl angles ranging from 104.84 (4)–114.54 (3)°.

## Supra­molecular features   

**Figure 2:**
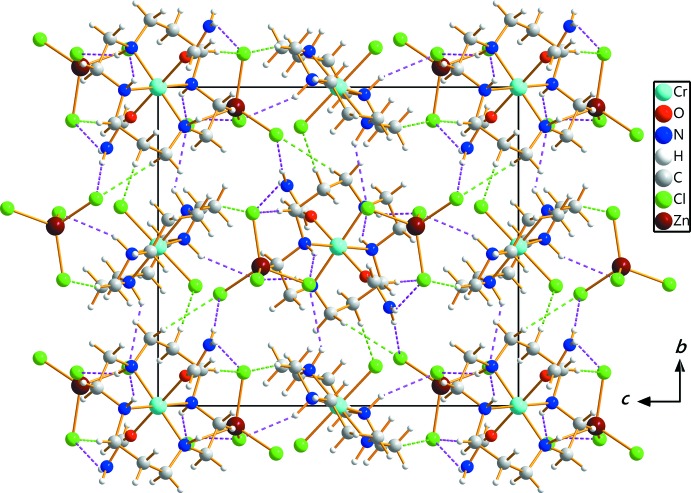
Crystal packing of (I), viewed along the *a* axis. Dashed lines represent hydrogen-bonding inter­actions [N—H⋯Cl (pink) and C—H⋯Cl (green)].

Extensive C—H⋯Cl and N–H⋯Cl hydrogen-bonding inter­actions occur between the NH or CH groups of cyclam and the NH_2_ group of formamide, the Cl ligand and the Cl atoms of the tetra­chloro­zincate anion (Table 1 ▸). The ZnCl_4_^2−^ anion is linked to two [CrCl_2_(cyclam)]^+^ and [Cr(fa)(cyclam)]^3+^ cations *via* a series of N—H⋯Cl and C—H⋯Cl hydrogen bonds. In addition, two Cr^III^ complex cations are inter­connected to each other *via* a C3—H3*A*⋯Cl1^vi^ [symmetry code: (vi) −*x* + 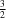, *y* + 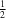, −*z* + 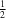] hydrogen bond. The extensive array of these contacts generates a three-dimensional network and helps to consolidate the crystal structure. The crystal packing diagram of (I) viewed perpendicular to the *bc* plane is shown in Fig. 2 ▸.

## Database survey   

A search of the Cambridge Structural Database (CSD, Version 5.41, November 2019; Groom *et al.*, 2016 ▸) indicated 76 hits for a [Cr*L*_2_(C_10_H_24_N_4_)]^*n*+^ unit. More than 30 different ligand types *L* including halogenides, cyanide, azide, thio­cyanate, oxalate, ammonia, sulfate, nitrite, DMSO and esters have been reported. It has been found that *trans*-[Cr(NCS)_2_(C_10_H_24_N_4_)]ClO_4_ (RAVGEA; Friesen *et al.*, 1997 ▸), *trans*-[Cr(nic-O)_2_(C_10_H_24_N_4_)]ClO_4_ (NUKMUC; Choi, 2009 ▸) and *trans*-[Cr(ONO)_2_)(C_10_H_24_N_4_)]BF_4_ (MEMHAN; De Leo *et al.*, 2000 ▸) adopt the *trans*-III conformations. On the other hand, *cis*-[Cr(NCS)_2_(C_10_H_24_N_4_)]ClO_4_ (RAVGOK; Friesen *et al.*, 1997 ▸), [Cr(C_2_O_4_)(C_10_H_24_N_4_)]ClO_4_ (IHAFOM; Choi *et al.*, 2004*b* ▸), [Cr(CH_3_COCHCOCH_3_)(C_10_H_24_N_4_)](ClO_4_)_2_·0.5H_2_O (SAYSES; Subhan *et al.*, 2011 ▸) and *cis*-[Cr(NCS)_2_(C_10_H_24_N_4_)]NCS (ADUXOO; Moon *et al.*, 2013 ▸) have the folded *cis*-V conformations. A search of the CSD gave 698 hits for cyclam (C_10_H_24_N_4_) with any metal but no hit for uncomplexed cyclam. In addition, no compounds containing [Cr(HCONH_2_)_2_(C_10_H_24_N_4_)]^3+^ were known until now.

## Synthesis and crystallization   

The free ligand cyclam and formamide were purchased from Sigma–Aldrich. The formamide was purified and dried by standard methods. All other chemicals were reagent-grade materials and used without further purification. The starting material, *trans*-[Cr(CN)_2_(cyclam)]ClO_4_, was prepared according to the literature (Kane-Maguire *et al.*, 1983 ▸). The yellow solid, *trans*-[Cr(CN)_2_(cyclam)]ClO_4_ (0.08 g) was dissolved in 5 mL of 0.01 *M* HCl, and heated for 2 h at 333 K. The solution was added to 3 mL of 6 *M* HCl containing 0.2 g of solid ZnCl_2_, and then 2 mL of formamide were added dropwise under magnetic stirring. The resulting solution was filtered, and allowed to stand at room temperature for a few weeks to give purple crystals of (I) suitable for X-ray structural analysis.

## Refinement   

Crystal data, data collection and structure refinement details are summarized in Table 2 ▸. All H atoms were placed in geometrically idealized positions and constrained to ride on their parent atoms, with C—H = 0.94–0.98 Å and N—H = 0.87–0.99 Å and with *U*_iso_(H) = 1.2*U*_eq_(C,N).

## Supplementary Material

Crystal structure: contains datablock(s) I. DOI: 10.1107/S2056989020004910/vm2231sup1.cif

Structure factors: contains datablock(s) I. DOI: 10.1107/S2056989020004910/vm2231Isup2.hkl

CCDC reference: 1995114

Additional supporting information:  crystallographic information; 3D view; checkCIF report

## Figures and Tables

**Table 1:** Hydrogen-bond geometry (Å, °)

*D*—H⋯*A*	*D*—H	H⋯*A*	*D*⋯*A*	*D*—H⋯*A*
N1—H1⋯Cl4^i^	0.99	2.46	3.346 (2)	149
N2—H2⋯Cl3	0.99	2.31	3.255 (2)	159
N3—H3*AN*⋯Cl5^ii^	0.87	2.65	3.505 (3)	167
N3—H3*BN*⋯Cl2^iii^	0.87	2.61	3.334 (3)	141
C2—H2*A*⋯Cl2^i^	0.98	2.65	3.606 (3)	165
N4—H4⋯Cl3^iv^	0.99	2.56	3.493 (2)	157
N5—H5⋯Cl4^v^	0.99	2.76	3.549 (2)	137
C3—H3*A*⋯Cl1^vi^	0.98	2.71	3.650 (3)	160
C4—H4*A*⋯Cl5^ii^	0.98	2.78	3.555 (3)	136
C7—H7*AB*⋯Cl2^iv^	0.98	2.81	3.738 (3)	159

Symmetry codes: (i) 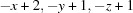; (ii) 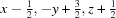; (iii) 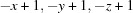; (iv) 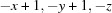; (v) 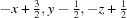; (vi) 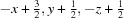.

**Table 2:** Experimental details

Crystal data	
Chemical formula	[CrCl_2_(C_10_H_24_N_4_)][Cr(CH_3_NO)_2_(C_10_H_24_N_4_)][ZnCl_4_]_2_
*M*_r_	1079.99
Crystal system, space group	Monoclinic, *P*2_1_/*n*
Temperature (K)	220
*a*, *b*, *c* (Å)	10.406 (2), 13.212 (3), 15.011 (3)
β (°)	95.85 (3)
*V* (Å^3^)	2053.0 (7)
*Z*	2
Radiation type	Synchrotron, λ = 0.610 Å
μ (mm^−1^)	1.53
Crystal size (mm)	0.13 × 0.11 × 0.08

Data collection	
Diffractometer	Rayonix MX225HS CCD area detector
Absorption correction	Empirical (using intensity measurements) (*HKL3000sm *SCALEPACK**; Otwinowski & Minor, 1997 ▸)
*T*_min_, *T*_max_	0.856, 1.000
No. of measured, independent and observed [*I* > 2σ(*I*)] reflections	20797, 5718, 5424
*R*_int_	0.065
(sin θ/λ)_max_ (Å^−1^)	0.693

Refinement	
*R*[*F* ^2^ > 2σ(*F* ^2^)], *wR*(*F* ^2^), *S*	0.042, 0.120, 1.08
No. of reflections	5718
No. of parameters	220
H-atom treatment	H-atom parameters constrained
Δρ_max_, Δρ_min_ (e Å^−3^)	1.02, −1.05

Computer programs: *PAL BL2D-SMDC* (Shin *et al.*, 2016 ▸), *HKL3000sm* (Otwinowski & Minor, 1997 ▸), *SHELXT2018* (Sheldrick, 2015*a* ▸), *SHELXL2018* (Sheldrick, 2015*b* ▸), *DIAMOND 4* (Putz & Brandenburg, 2014 ▸) and *publCIF* (Westrip, 2010 ▸).

## supplementary crystallographic information

### Crystal data

**Table:**

[CrCl_2_(C_10_H_24_N_4_)][Cr(CH_3_NO)_2_(C_10_H_24_N_4_)][ZnCl_4_]_2_	*F*(000) = 1100
*M**_r_* = 1079.99	*D*_x_ = 1.747 Mg m^−^^3^
Monoclinic, *P*2_1_/*n*	Synchrotron radiation, λ = 0.610 Å
*a* = 10.406 (2) Å	Cell parameters from 71380 reflections
*b* = 13.212 (3) Å	θ = 0.4–33.7°
*c* = 15.011 (3) Å	µ = 1.53 mm^−^^1^
β = 95.85 (3)°	*T* = 220 K
*V* = 2053.0 (7) Å^3^	Plate, purple
*Z* = 2	0.13 × 0.11 × 0.08 mm

### Data collection

**Table:**

Rayonix MX225HS CCD area detector diffractometer	5424 reflections with *I* > 2σ(*I*)
Radiation source: PLSII 2D bending magnet	*R*_int_ = 0.065
ω scan	θ_max_ = 25.0°, θ_min_ = 1.8°
Absorption correction: empirical (using intensity measurements) (*HKL3000sm Scalepack*; Otwinowski & Minor, 1997)	*h* = −14→14
*T*_min_ = 0.856, *T*_max_ = 1.000	*k* = −18→18
20797 measured reflections	*l* = −20→20
5718 independent reflections	

### Refinement

**Table:**

Refinement on *F*^2^	0 restraints
Least-squares matrix: full	Hydrogen site location: inferred from neighbouring sites
*R*[*F*^2^ > 2σ(*F*^2^)] = 0.042	H-atom parameters constrained
*wR*(*F*^2^) = 0.120	*w* = 1/[σ^2^(*F*_o_^2^) + (0.0579*P*)^2^ + 2.3444*P*] where *P* = (*F*_o_^2^ + 2*F*_c_^2^)/3
*S* = 1.08	(Δ/σ)_max_ = 0.001
5718 reflections	Δρ_max_ = 1.02 e Å^−^^3^
220 parameters	Δρ_min_ = −1.05 e Å^−^^3^

### Special details

**Table:**

Geometry. All esds (except the esd in the dihedral angle between two l.s. planes) are estimated using the full covariance matrix. The cell esds are taken into account individually in the estimation of esds in distances, angles and torsion angles; correlations between esds in cell parameters are only used when they are defined by crystal symmetry. An approximate (isotropic) treatment of cell esds is used for estimating esds involving l.s. planes.

### Fractional atomic coordinates and isotropic or equivalent isotropic displacement parameters (Å^2^)

**Table:**

	*x*	*y*	*z*	*U*_iso_*/*U*_eq_	
Cr1	0.500000	0.500000	0.500000	0.01905 (11)	
O1	0.40779 (18)	0.59415 (14)	0.57570 (12)	0.0304 (4)	
N1	0.6599 (2)	0.50926 (16)	0.59314 (13)	0.0263 (4)	
H1	0.733309	0.478812	0.565654	0.032*	
N2	0.5477 (2)	0.62362 (15)	0.42632 (14)	0.0270 (4)	
H2	0.612781	0.600675	0.387054	0.032*	
N3	0.2731 (3)	0.6910 (2)	0.64216 (15)	0.0381 (5)	
H3AN	0.332356	0.736628	0.655071	0.046*	
H3BN	0.195781	0.699839	0.657996	0.046*	
C1	0.6336 (3)	0.4436 (2)	0.66990 (16)	0.0335 (5)	
H1A	0.576230	0.478762	0.707614	0.040*	
H1AB	0.714487	0.427910	0.706529	0.040*	
C2	0.6985 (3)	0.6141 (2)	0.62080 (19)	0.0352 (6)	
H2A	0.776779	0.611563	0.662933	0.042*	
H2AB	0.629729	0.645040	0.651654	0.042*	
C3	0.7237 (3)	0.6793 (2)	0.5405 (2)	0.0399 (6)	
H3A	0.768805	0.740799	0.562773	0.048*	
H3AB	0.781941	0.642161	0.504919	0.048*	
C4	0.6046 (3)	0.7105 (2)	0.4789 (2)	0.0362 (6)	
H4A	0.539889	0.738832	0.514956	0.043*	
H4AB	0.628599	0.763302	0.437894	0.043*	
C5	0.4294 (3)	0.6526 (2)	0.36674 (17)	0.0343 (5)	
H5A	0.452908	0.695465	0.317621	0.041*	
H5AB	0.369790	0.690444	0.400752	0.041*	
C6	0.3001 (3)	0.60960 (19)	0.59923 (15)	0.0280 (5)	
H6	0.235060	0.561016	0.585794	0.034*	
Cr2	0.500000	0.500000	0.000000	0.02063 (12)	
Cl1	0.53785 (6)	0.37596 (5)	−0.10350 (4)	0.03271 (14)	
N4	0.3223 (2)	0.52342 (16)	−0.07214 (13)	0.0249 (4)	
H4	0.312441	0.470207	−0.118767	0.030*	
N5	0.4335 (2)	0.38790 (16)	0.08048 (13)	0.0259 (4)	
H5	0.431725	0.324363	0.045410	0.031*	
C7	0.3340 (2)	0.62148 (19)	−0.11970 (16)	0.0289 (5)	
H7A	0.326491	0.677919	−0.078176	0.035*	
H7AB	0.264587	0.627579	−0.168695	0.035*	
C8	0.2067 (2)	0.5165 (2)	−0.02153 (17)	0.0307 (5)	
H8A	0.128505	0.524049	−0.063273	0.037*	
H8AB	0.208634	0.572239	0.021703	0.037*	
C9	0.2009 (3)	0.4161 (2)	0.02812 (19)	0.0342 (5)	
H9A	0.210352	0.361121	−0.014533	0.041*	
H9AB	0.114986	0.409655	0.048899	0.041*	
C10	0.3022 (3)	0.4014 (2)	0.10863 (17)	0.0319 (5)	
H10A	0.301640	0.460400	0.148121	0.038*	
H10B	0.279482	0.341775	0.142624	0.038*	
C11	0.5356 (3)	0.3751 (2)	0.15662 (16)	0.0297 (5)	
H11A	0.524570	0.310127	0.186461	0.036*	
H11B	0.529597	0.429393	0.200469	0.036*	
Zn1	0.96538 (3)	0.56371 (2)	0.28510 (2)	0.02620 (9)	
Cl2	0.98118 (7)	0.39522 (5)	0.26256 (6)	0.04267 (17)	
Cl3	0.74908 (6)	0.60423 (6)	0.27489 (4)	0.03418 (15)	
Cl4	1.06521 (7)	0.61932 (6)	0.41833 (4)	0.03892 (16)	
Cl5	1.04313 (8)	0.64946 (6)	0.17226 (5)	0.04333 (17)	

### Atomic displacement parameters (Å^2^)

**Table:**

	*U*^11^	*U*^22^	*U*^33^	*U*^12^	*U*^13^	*U*^23^
Cr1	0.0141 (2)	0.0235 (2)	0.0203 (2)	−0.00100 (17)	0.00503 (17)	−0.00244 (16)
O1	0.0269 (9)	0.0323 (8)	0.0318 (8)	−0.0007 (7)	0.0021 (7)	−0.0048 (7)
N1	0.0193 (9)	0.0342 (10)	0.0252 (9)	0.0019 (8)	0.0023 (7)	−0.0054 (7)
N2	0.0258 (9)	0.0278 (9)	0.0290 (9)	−0.0009 (8)	0.0112 (8)	−0.0015 (7)
N3	0.0382 (12)	0.0437 (12)	0.0337 (11)	0.0089 (11)	0.0106 (9)	−0.0063 (9)
C1	0.0319 (13)	0.0465 (14)	0.0219 (10)	0.0058 (11)	0.0012 (9)	−0.0015 (9)
C2	0.0252 (12)	0.0393 (13)	0.0396 (13)	−0.0027 (11)	−0.0040 (10)	−0.0118 (11)
C3	0.0270 (12)	0.0382 (13)	0.0551 (16)	−0.0124 (11)	0.0073 (12)	−0.0071 (12)
C4	0.0354 (14)	0.0298 (11)	0.0448 (14)	−0.0075 (11)	0.0120 (11)	−0.0030 (10)
C5	0.0367 (13)	0.0367 (12)	0.0302 (11)	0.0044 (11)	0.0076 (10)	0.0056 (10)
C6	0.0286 (11)	0.0343 (11)	0.0216 (9)	0.0002 (10)	0.0045 (8)	0.0011 (8)
Cr2	0.0185 (2)	0.0252 (2)	0.0181 (2)	0.00430 (18)	0.00144 (18)	−0.00252 (16)
Cl1	0.0330 (3)	0.0374 (3)	0.0274 (3)	0.0097 (3)	0.0014 (2)	−0.0065 (2)
N4	0.0218 (9)	0.0307 (9)	0.0218 (8)	0.0056 (8)	0.0000 (7)	−0.0043 (7)
N5	0.0242 (9)	0.0311 (9)	0.0224 (8)	0.0015 (8)	0.0022 (7)	−0.0003 (7)
C7	0.0258 (11)	0.0338 (11)	0.0264 (10)	0.0108 (10)	−0.0003 (8)	0.0010 (9)
C8	0.0189 (10)	0.0417 (13)	0.0313 (11)	0.0054 (10)	0.0024 (9)	−0.0029 (10)
C9	0.0216 (11)	0.0435 (13)	0.0375 (13)	−0.0062 (11)	0.0029 (10)	−0.0013 (11)
C10	0.0261 (11)	0.0406 (13)	0.0297 (11)	−0.0020 (10)	0.0066 (9)	0.0008 (10)
C11	0.0301 (12)	0.0351 (12)	0.0235 (10)	0.0041 (10)	0.0011 (9)	0.0028 (9)
Zn1	0.02112 (15)	0.03240 (16)	0.02530 (14)	0.00089 (11)	0.00344 (11)	0.00152 (10)
Cl2	0.0293 (3)	0.0332 (3)	0.0636 (4)	0.0034 (3)	−0.0047 (3)	−0.0042 (3)
Cl3	0.0233 (3)	0.0487 (4)	0.0312 (3)	0.0076 (3)	0.0060 (2)	0.0028 (2)
Cl4	0.0373 (3)	0.0493 (4)	0.0288 (3)	0.0094 (3)	−0.0033 (2)	−0.0059 (3)
Cl5	0.0461 (4)	0.0509 (4)	0.0353 (3)	−0.0029 (3)	0.0155 (3)	0.0100 (3)

### Geometric parameters (Å, º)

**Table:**

Cr1—O1	1.9953 (19)	Cr2—N4	2.069 (2)
Cr1—O1^i^	1.9954 (19)	Cr2—N4^ii^	2.069 (2)
Cr1—N2	2.061 (2)	Cr2—N5^ii^	2.074 (2)
Cr1—N2^i^	2.061 (2)	Cr2—N5	2.074 (2)
Cr1—N1^i^	2.065 (2)	Cr2—Cl1	2.3194 (7)
Cr1—N1	2.065 (2)	Cr2—Cl1^ii^	2.3194 (7)
O1—C6	1.226 (3)	N4—C8	1.490 (3)
N1—C1	1.489 (3)	N4—C7	1.490 (3)
N1—C2	1.490 (3)	N4—H4	0.9900
N1—H1	0.9900	N5—C10	1.482 (3)
N2—C4	1.482 (3)	N5—C11	1.488 (3)
N2—C5	1.495 (3)	N5—H5	0.9900
N2—H2	0.9900	C7—C11^ii^	1.519 (4)
N3—C6	1.299 (3)	C7—H7A	0.9800
N3—H3AN	0.8700	C7—H7AB	0.9800
N3—H3BN	0.8700	C8—C9	1.526 (4)
C1—C5^i^	1.509 (4)	C8—H8A	0.9800
C1—H1A	0.9800	C8—H8AB	0.9800
C1—H1AB	0.9800	C9—C10	1.533 (4)
C2—C3	1.525 (4)	C9—H9A	0.9800
C2—H2A	0.9800	C9—H9AB	0.9800
C2—H2AB	0.9800	C10—H10A	0.9800
C3—C4	1.525 (4)	C10—H10B	0.9800
C3—H3A	0.9800	C11—H11A	0.9800
C3—H3AB	0.9800	C11—H11B	0.9800
C4—H4A	0.9800	Zn1—Cl5	2.2555 (8)
C4—H4AB	0.9800	Zn1—Cl2	2.2602 (9)
C5—H5A	0.9800	Zn1—Cl4	2.2792 (9)
C5—H5AB	0.9800	Zn1—Cl3	2.3035 (8)
C6—H6	0.9400		
			
O1—Cr1—O1^i^	180.0	N4—Cr2—N4^ii^	180.0
O1—Cr1—N2	88.14 (8)	N4—Cr2—N5^ii^	85.50 (8)
O1^i^—Cr1—N2	91.86 (8)	N4^ii^—Cr2—N5^ii^	94.49 (8)
O1—Cr1—N2^i^	91.86 (8)	N4—Cr2—N5	94.49 (8)
O1^i^—Cr1—N2^i^	88.14 (8)	N4^ii^—Cr2—N5	85.51 (8)
N2—Cr1—N2^i^	180.00 (7)	N5^ii^—Cr2—N5	180.0
O1—Cr1—N1^i^	91.23 (8)	N4—Cr2—Cl1	87.64 (6)
O1^i^—Cr1—N1^i^	88.77 (8)	N4^ii^—Cr2—Cl1	92.36 (6)
N2—Cr1—N1^i^	84.54 (9)	N5^ii^—Cr2—Cl1	91.43 (6)
N2^i^—Cr1—N1^i^	95.46 (9)	N5—Cr2—Cl1	88.57 (6)
O1—Cr1—N1	88.77 (8)	N4—Cr2—Cl1^ii^	92.36 (6)
O1^i^—Cr1—N1	91.23 (8)	N4^ii^—Cr2—Cl1^ii^	87.64 (6)
N2—Cr1—N1	95.45 (9)	N5^ii^—Cr2—Cl1^ii^	88.57 (6)
N2^i^—Cr1—N1	84.54 (9)	N5—Cr2—Cl1^ii^	91.43 (6)
N1^i^—Cr1—N1	180.0	Cl1—Cr2—Cl1^ii^	180.0
C6—O1—Cr1	140.79 (18)	C8—N4—C7	114.01 (19)
C1—N1—C2	113.0 (2)	C8—N4—Cr2	116.69 (15)
C1—N1—Cr1	106.88 (16)	C7—N4—Cr2	105.58 (15)
C2—N1—Cr1	114.80 (16)	C8—N4—H4	106.6
C1—N1—H1	107.3	C7—N4—H4	106.6
C2—N1—H1	107.3	Cr2—N4—H4	106.6
Cr1—N1—H1	107.3	C10—N5—C11	113.65 (19)
C4—N2—C5	112.3 (2)	C10—N5—Cr2	116.90 (16)
C4—N2—Cr1	115.64 (16)	C11—N5—Cr2	105.93 (15)
C5—N2—Cr1	107.20 (15)	C10—N5—H5	106.6
C4—N2—H2	107.1	C11—N5—H5	106.6
C5—N2—H2	107.1	Cr2—N5—H5	106.6
Cr1—N2—H2	107.1	N4—C7—C11^ii^	108.69 (19)
C6—N3—H3AN	120.0	N4—C7—H7A	110.0
C6—N3—H3BN	120.0	C11^ii^—C7—H7A	110.0
H3AN—N3—H3BN	120.0	N4—C7—H7AB	110.0
N1—C1—C5^i^	108.4 (2)	C11^ii^—C7—H7AB	110.0
N1—C1—H1A	110.0	H7A—C7—H7AB	108.3
C5^i^—C1—H1A	110.0	N4—C8—C9	112.1 (2)
N1—C1—H1AB	110.0	N4—C8—H8A	109.2
C5^i^—C1—H1AB	110.0	C9—C8—H8A	109.2
H1A—C1—H1AB	108.4	N4—C8—H8AB	109.2
N1—C2—C3	111.6 (2)	C9—C8—H8AB	109.2
N1—C2—H2A	109.3	H8A—C8—H8AB	107.9
C3—C2—H2A	109.3	C8—C9—C10	115.9 (2)
N1—C2—H2AB	109.3	C8—C9—H9A	108.3
C3—C2—H2AB	109.3	C10—C9—H9A	108.3
H2A—C2—H2AB	108.0	C8—C9—H9AB	108.3
C4—C3—C2	115.9 (2)	C10—C9—H9AB	108.3
C4—C3—H3A	108.3	H9A—C9—H9AB	107.4
C2—C3—H3A	108.3	N5—C10—C9	111.7 (2)
C4—C3—H3AB	108.3	N5—C10—H10A	109.3
C2—C3—H3AB	108.3	C9—C10—H10A	109.3
H3A—C3—H3AB	107.4	N5—C10—H10B	109.3
N2—C4—C3	111.7 (2)	C9—C10—H10B	109.3
N2—C4—H4A	109.3	H10A—C10—H10B	107.9
C3—C4—H4A	109.3	N5—C11—C7^ii^	108.09 (19)
N2—C4—H4AB	109.3	N5—C11—H11A	110.1
C3—C4—H4AB	109.3	C7^ii^—C11—H11A	110.1
H4A—C4—H4AB	107.9	N5—C11—H11B	110.1
N2—C5—C1^i^	107.6 (2)	C7^ii^—C11—H11B	110.1
N2—C5—H5A	110.2	H11A—C11—H11B	108.4
C1^i^—C5—H5A	110.2	Cl5—Zn1—Cl2	110.19 (3)
N2—C5—H5AB	110.2	Cl5—Zn1—Cl4	109.31 (3)
C1^i^—C5—H5AB	110.2	Cl2—Zn1—Cl4	114.54 (3)
H5A—C5—H5AB	108.5	Cl5—Zn1—Cl3	104.84 (4)
O1—C6—N3	122.1 (3)	Cl2—Zn1—Cl3	107.73 (3)
O1—C6—H6	118.9	Cl4—Zn1—Cl3	109.78 (4)
N3—C6—H6	118.9		
			
C2—N1—C1—C5^i^	168.9 (2)	C8—N4—C7—C11^ii^	171.66 (19)
Cr1—N1—C1—C5^i^	41.6 (2)	Cr2—N4—C7—C11^ii^	42.3 (2)
C1—N1—C2—C3	−179.6 (2)	C7—N4—C8—C9	−178.4 (2)
Cr1—N1—C2—C3	−56.6 (3)	Cr2—N4—C8—C9	−54.8 (2)
N1—C2—C3—C4	72.1 (3)	N4—C8—C9—C10	70.3 (3)
C5—N2—C4—C3	179.0 (2)	C11—N5—C10—C9	179.0 (2)
Cr1—N2—C4—C3	55.5 (3)	Cr2—N5—C10—C9	55.1 (3)
C2—C3—C4—N2	−71.2 (3)	C8—C9—C10—N5	−70.4 (3)
C4—N2—C5—C1^i^	−169.7 (2)	C10—N5—C11—C7^ii^	−171.3 (2)
Cr1—N2—C5—C1^i^	−41.6 (2)	Cr2—N5—C11—C7^ii^	−41.7 (2)
Cr1—O1—C6—N3	−170.2 (2)		

Symmetry codes: (i) −*x*+1, −*y*+1, −*z*+1; (ii) −*x*+1, −*y*+1, −*z*.

### Hydrogen-bond geometry (Å, º)

**Table:**

*D*—H···*A*	*D*—H	H···*A*	*D*···*A*	*D*—H···*A*
N1—H1···Cl4^iii^	0.99	2.46	3.346 (2)	149
N2—H2···Cl3	0.99	2.31	3.255 (2)	159
N3—H3*AN*···Cl5^iv^	0.87	2.65	3.505 (3)	167
N3—H3*BN*···Cl2^i^	0.87	2.61	3.334 (3)	141
C2—H2*A*···Cl2^iii^	0.98	2.65	3.606 (3)	165
N4—H4···Cl3^ii^	0.99	2.56	3.493 (2)	157
N5—H5···Cl4^v^	0.99	2.76	3.549 (2)	137
C3—H3*A*···Cl1^vi^	0.98	2.71	3.650 (3)	160
C4—H4*A*···Cl5^iv^	0.98	2.78	3.555 (3)	136
C7—H7*AB*···Cl2^ii^	0.98	2.81	3.738 (3)	159

Symmetry codes: (i) −*x*+1, −*y*+1, −*z*+1; (ii) −*x*+1, −*y*+1, −*z*; (iii) −*x*+2, −*y*+1, −*z*+1; (iv) *x*−1/2, −*y*+3/2, *z*+1/2; (v) −*x*+3/2, *y*−1/2, −*z*+1/2; (vi) −*x*+3/2, *y*+1/2, −*z*+1/2.
